# An Examination of Subway Sex Offense Modus Operandi: A Case of Seoul, South Korea

**DOI:** 10.3390/ijerph17238979

**Published:** 2020-12-02

**Authors:** Taeyoung Kim, Seung Yeop Paek, Julak Lee

**Affiliations:** 1 Security Management Institute, 527, Eonju-ro, Gangnam-gu, Seoul 06138, Korea; kty57@hanmail.net; 2 Department of Criminal Justice, California State University, East Bay, Hayward, CA 94542, USA; seung.paek@csueastbay.edu; 3 Department of Industrial Security, Chung-Ang University, Seoul 06974, Korea

**Keywords:** subway sex offenses, modus operandi, crime script analysis, sexual harassment, surreptitious recording

## Abstract

Subway sex offenses are a serious issue in the nations around world, but existing research has failed to explore the offense types or patterns systematically. In order to fill this gap, the authors employed Crime Script Analysis (CSA) to examine the two most common subway sex offenses in Seoul, South Korea. Specifically, the authors assessed the reasoning behind the steps taken to prepare for, carry out, and complete harassment and surreptitious recording. The offenses committed in the subway stations around the city of Seoul were analyzed based on the interviews with the subway police, official crime reports, and crime case files. Drawing from the findings, theoretical and practical implications are discussed.

## 1. Introduction

The subway systems around the world, especially in large cities, have developed and expanded with time. The change has often promoted beneficial effects on the surrounding communities by increasing the property values and vitalizing the local economy [1,2,3]. However, subway crimes have also evolved and accompanied such advancement. In the case of Seoul, South Korea, the Seoul Metro which is one of the world’s largest railway networks with a total of nine lines and 293 stations carries more than seven million citizens each day, offering numerous criminal opportunities for sex offenders [4,5]. Common types of subway crimes in South Korea include larceny-theft, assault, and sex offense, of which sex crimes constitute more than 40% of the offenses committed in Seoul [6].

Sex offenses could have serious impact on the victims’ lives. The victims of the sex offenses committed in public transportation are prone to increased anxiety and fear of crime, decreased self-esteem [7,8,9] as well as avoid particular times or modes of transportation to decrease further victimization [10,11]. Considering that personal security and health are basic human needs [12], perception of victimization and fear of crime could negatively affect the victims’ quality of life.

Particularly, female passengers who face higher likelihood of becoming a target of sexual violence in public spaces have expressed concern about using public transportation [13,14,15]. For example, the “2018 Seoul Gender Sensitivity Statistics” published by Seoul Foundation of Women and Family reports that around 71.9% of women are fearful of victimization in the subway [16].

Despite the implication for the safety of passengers, there has been a lack of systematic approach to understand and root out the underlying problems of the subway sex crimes in South Korea. The measures including installation of closed-circuit television (CCTV) and creation of women-only passenger cars have been suggested to be fragmented responses with no existing evidence supporting their effectiveness [17]. Therefore, an in-depth analysis on the nature and the characteristics of the offenses that could lead to viable prevention and response programs is warranted.

The current research investigates the state and characteristics of the sex crimes committed in the subway stations in Seoul, South Korea with the purpose to explore offender behavioral patterns in each step of the crime-commission process and develop a generalizable offending modus operandi by using Crime Script Analysis (CSA) [18]. Specifically, the authors attempt to answer the following research questions: “What decisions are made by the offenders before, during, and after crime commission?; what are the situational factors that affect subway sex offense victimization?”; and “what strategies do offenders employ to increase success and minimize failure?”

## 2. Literature Review

### 2.1. Subway Sex Offenses

Sexual offense in public spaces is an issue faced by many nations around the world [19,20]. Although researchers have examined sexual harassment in India [15,21], Mexico [22], and Nepal [23], the offenses committed in the cars and the facilities built in and around the subway stations, including the entrances and exits, the platforms, and the passages [24] have not been researched as extensively.

Subway stations are replete with criminal opportunities, making them an attractive crime location from the rational choice and routine activities perspectives. Routine activities theory (RAT) [25] suggests that criminal victimization is likely when a motivated offender, a suitable target, and the absence of a capable guardian converge in time and place. The unique nature of the physical environment of the subway (i.e., underground) as well as crowded cars and passages increase the number of suitable targets and undermine the presence of capable guardians, making it difficult to control the illicit activities [17,26].

As stated, the two common sex crimes in the Seoul subway are harassment and surreptitious recording which can be classified into frotteurism and voyeurism, respectively. The former refers to a paraphilia in which a person seeks sexual pleasure by touching and rubbing against another person, and the latter by observing others that are naked, in the act of undressing, or engaging in sexual activity [27,28]. These non-contact crimes that do not involve penetration have been traditionally considered as nuisance offenses [29,30], and the passing and enactment of relevant laws have lagged behind [14].

Before the advent and development of digital equipment, sexual harassment constituted the vast majority of subway sex offenses. However, technology has diversified and facilitated the offenses committed with advanced tools [14]. Particularly, surreptitious recording has become one of the most serious issues in the South Korean subway with the Internet providing a platform to exchange sexually explicit material in cyberspace [6]. As Table 1 shows, surreptitious recording comprised about thirty-one percent of all reported subway sex offense incidents in 2019 (378 out of 1,206). The actual prevalence of the sex offenses is expected to be higher due to underreporting and unknown victimization [31,32].

Though additional investigation on female sexual victimization in transit environments is needed [33], a line of relevant research has emphasized gender differences in travel pattern, victimization, and fear of crime. For instance, Smith [34] has argued that the specific needs of women passengers must be taken into account by the authorities. Similarly, a study conducted on female college students’ sexual victimization in public transport suggests that victimization risks are prevalent throughout their commute to school [32]. From the feminist perspective, the issue of sexual violence in transit environment is interpreted as a form of social injustice and patriarchy that impedes women’s autonomy and mobility [35,36]. Nonetheless, it has been found that there are few existing programs that help meet women’s unique travel needs, prompting researchers to argue that the government must develop and implement programs to alleviate women passengers’ fear while using public transportation [9,34]. It is further noted that failing to provide safe travel environments for women and thus restricting their mobility constitutes an infringement of their basic rights [19] and perpetuates gender inequality in our society [37].

In non-Western settings, Ceccato and Paz [38] used geographical information system (GIS) to find that the sex offenses committed in São Paulo Metro were concentrated in the most crowded areas and during the rush hours. Furthermore, because female passengers report greater fear of victimization in public transportation compared to men [37], measures to alleviate the concerns about victimization, including women-only subway passenger cars have emerged in countries such as Japan [8].

In the cultural context of South Korea, researchers have focused on effective response through enhanced policing strategies (e.g., [39]). To illustrate, modifying the physical environments (i.e. Crime Prevention Through Environmental Design or CPTED) [40] and reducing criminal opportunities by increasing police presence and awareness on the issue of victim blaming have been suggested as ways to prevent and reduce the impact of sex crimes [4,31,41]. Taking a similar approach by calling for increased visibility and improved surveillance of the spaces in the subway stations, Roh and Kim [42] have suggested multi-agency partnerships for effective control of the subway crimes.

A review of the literature shows that focused analysis of situation-specific offender modus operandi is virtually nonexistent. Though informing, most studies have referred to the official statistics as the basis for policy recommendations. In order to fill this gap, the current research explores the specific features and strategies of sexual harassment and surreptitious recording in the subway of Seoul, South Korea. Aside from the official statistics, the CSA is conducted based on the interviews with police officers and the crime case files.

### 2.2. Crime Script Analysis

The rational choice perspective argues that offenders commit crimes based on the perception that the benefits (e.g., desired goods, sexual gratification, money, etc.) outweigh the costs (e.g., legal sanctions, disreputation, etc.). Specific methods and decisions involved in the crime-commission process can also be evaluated from the same viewpoint as the offender behaviors are restricted by available resources, time, and ability [43,44].

In order to enhance the understanding of the offending decisions and patterns throughout the whole crime-commission process, Cornish [18,45] borrowed from cognitive psychology a concept of “schema” which can be defined as a collection of knowledge about an entity that servs as a guide to perception or problem solving [46] and introduced the idea of crime script. These scripts which are acquired through social learning elaborate the procedures involved in the entire crime-commission process [47,48]. Specifically, the decisions made in each step of crime-commission process (before, during, and after) constitute the modus operandi of an offense [47]. Though offenders may improvise and adjust their actions if needed in order to successfully carry out the offending [18,45,47], the crime scripts serve as a template for future offending behavior once learned and undertaken, offering a useful guidance for crime prevention by helping to identify situational crime prevention points [45,49].

Due to its merits, CSA has been employed to understand various forms of crime, including trafficking of sex workers [50] and drug crimes [51], brokering in the stolen goods market [52], check fraud [53], sexual assaults [54,55], illegal landfill [56], stealing and selling data online [57], and voice phishing [58]. Furthermore, the existing studies have analyzed various types of data such as court manuscript [47], offender survey [59] and interview [60], and web forum conversations [57], demonstrating the utility and wide applicability of CSA. Particularly, it has been shown that CSA is an effective tool for an in-depth understanding of offender as well as victim profiling. The current research extends the use of CSA to the subway sex crimes in the cultural context of South Korea.

## 3. Methods

As discussed, the primary purpose of the current research was to explore the modus operandi of sexual harassment and surreptitious recording in the subway stations in Seoul, South Korea. The authors chose Seoul as the research site for the following reasons. As the capital, Seoul serves as the nation’s socio-political, economic, and social hub. According to the official statistics provided by Korean Statistical Information Service [61], 9,639,541 people reside in the city, nearly 19% of the nation’s total population (51,779,203). Additionally, the vast majority of the subway crimes are committed in the Seoul subway [6].

Unlike the studies that solely rely on one source of data (mostly offender interview or official statistics), official subway crime statistics, crime case files, and interviews with the subway police were analyzed. By triangulating data, the authors attempted to overcome the limitations of using single method and improve the validity of the findings [62].

Thirty-five subway crime cases managed by the Seoul Subway Police from January to March 2019 were analyzed. The case files included personal information about the offenders and the victims, how the offenses were carried out, and other data on the incidents that the authors used to infer the situational factors of the offenses. Due to the sensitive nature of the contents, the files were accessed at the agency after obtaining prior approval.

In addition, because the crime case files and the official statistics did not reflect the behavioral and psychological aspects of the offenders, in-depth interviews were conducted with the officers. The authors understood that interviewing the offenders directly would be an ideal way to examine their thoughts and the decision-making processes, but it was not a viable option because of the legal restrictions grounded on the “Personal Information Protection Act”. Nonetheless, the alternative method of consulting the officers were deemed sufficient enough to serve the research purpose.

A total of twelve male officers were interviewed. Participants included senior police officers, assistant inspectors, and inspectors who were in their 30s (5) or 40s (7). The selection criteria included more than one year of experience as a subway police officer as well as investigation or apprehension of more than ten sex offenders. Two officers had arrested at least 100 sex offenders and most of the others had apprehended 30 or more offenders, providing sufficient information to help answer the research questions.

The semi-structured interviewees were conducted from September to November, 2019, with each lasting from forty to seventy minutes. The interviews began with open-ended questions (i.e., the specific research questions presented on page two) covering the topics of offender types, behavioral patterns, and mental states, and the responses were recorded for further analysis. Moreover, the interview findings and the information from the criminal case files were cross-checked. The official statistics published by the KNPA [6] were also examined for a breakdown of the offenses by time. Collected data were analyzed by using content analysis which allowed the authors to identify the themes related to the research questions and explore the relationships between them [63].

## 4. Findings

### 4.1. Sexual Harassment

#### 4.1.1. Pre-Crime Stage

In preparation of sexual harassment, the offenders searched for a crowded passenger car, waited for the rush hour, selected an exit station, and looked for a suitable target. Mostly, sexual harassment occurred in a crowded car in which the passenger mobility was limited to a certain extent, increasing unintended and unwanted physical contacts. The offenders used this to their advantage and often targeted a stationary person who was easy to examine, approach, and harass.

**Figure 1:**
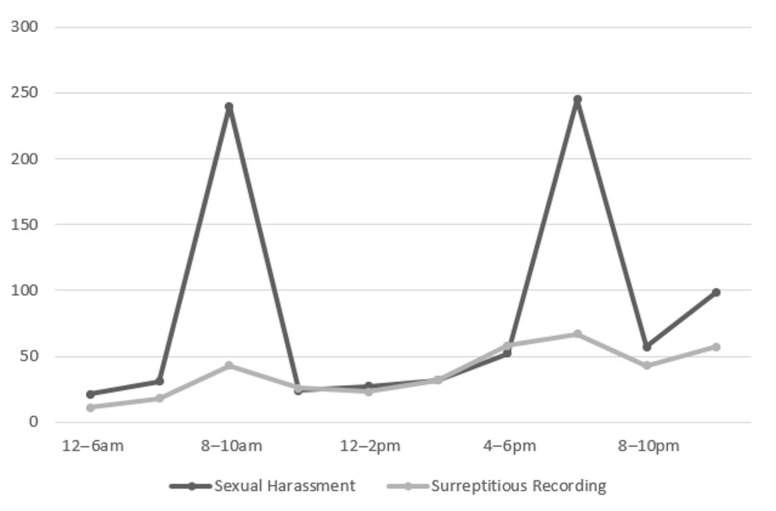
Number of Sexual Offense Occurrences by Time in 2019 [6].

The officers informed that the offenders preferred busy hours. Particularly, the morning (6–8 am) and the evening (6–8 pm) h during which the passenger cars were filled with commuters were known as the “hot times.” Sexual harassment incidents declined outside these time frames, though the numbers tended to climb again between 10 pm and midnight. The official data (Figure 1) validated the interview findings.

The interviewees also explained that the morning commute offered numerous opportunities and was the most favored time, followed by the after-work hours. It was reported that the night hours between 10 pm and midnight during which intoxicated and/or sleeping passengers increased attracted the harassers as well.

Moreover, the harassers planned for an exit after committing the crimes. They were found to take advantage of the moving trains and large numbers of passengers that boarded and disembarked them repeatedly between stations. Taking this pattern into account, the offenders calculated the timing of escape and walked out of the car using the shortest routes promptly after the doors opened.

As for target selection, there were some cases in which the harassers searched for a target to whom they were sexually attracted. One offender stalked a particular woman by appearing at the same station and waiting for the same car for around two months. Nonetheless, most offenders tended to wait for a suitable setting before selecting the target.

#### 4.1.2. Criminal Event Stage

Commission of sexual harassment was evaluated as a three-step process. Prior to boarding the train, offenders scanned the surrounding areas for potential police stakeouts using the reflections in the screen doors (installed on the subway platforms to prevent suicides). By doing so, the offenders avoided looking around and appearing suspicious. Once inside the car, the offenders checked the locations of the CCTV and attempted to spot police patrol activities. Crowded cars were preferred to empty ones, and when the targets boarded, the offenders pursued them persistently regardless of how dense the area was, especially if the victims were wearing short skirts or tight pants.

Next, the offenders stayed behind or stood face-to-face with the victim to facilitate making physical contacts, and hand-held bags, shopping bags, or coats were often used to cover their hands while committing the crime. The common modes of physical contacts included placing the back of a hand on the victim’s buttocks and thighs, blowing into the ears, and rubbing the genitals against the buttocks. If the victims did not react to the inappropriate contact, the offenders tended to become more aggressive while continuing the harassment. Another noteworthy harassment pattern identified was offenders staying and waiting closely behind the victims until the car arrived at the next station. Once the doors opened, the offenders touched either the buttocks or the chests of the victims and ran away.

#### 4.1.3. Post-Offense Stage

**Figure 2:**
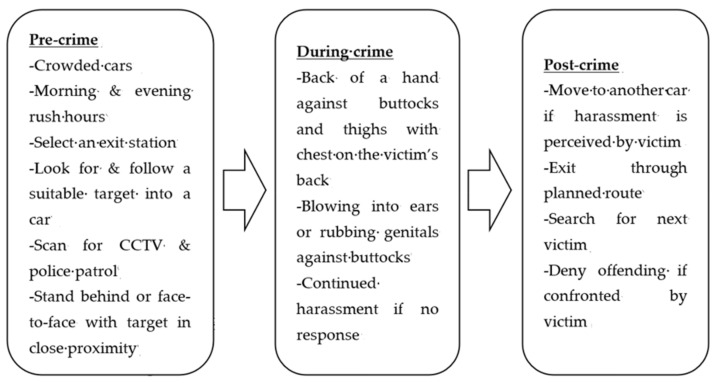
Sexual Harassment Crime Script.

In the post-offense stage, two main behavioral patterns were identified: retreat or deny if there was a reaction from the victim and searching for another target if harassment was completed without receiving any responses. The officers informed that the offenders escaped to a different car or alighted the train if they believed that the victim had perceived and reacted to their contacts assertively. If confronted by the victims, the offenders usually denied harassing them by stating that they were “law-abiding citizens” and played innocent, berated the victims for false accusation (often the elderly offenders), or ran away (i.e., hit-and-run). If there were no responses from the victims, sexual harassments were likely to continue and the offenders moved on to find the next target (Figure 2 summarizes the entire crime-commission process).

### 4.2. Surreptitious Recording

#### 4.2.1. Pre-Crime Stage

Five notable patterns were found in the pre-crime stage of surreptitious recording. First, the illicit photographing and filming incidents increased when temperature rose. Data revealed that surreptitious shooting incidents increased from February to September and decreased from October to January. The officers cited the prevalence of lighter clothing during warmer months as the main contributor to the copious opportunities for voyeurism.

Furthermore, it was found that the offenders waited for the evening rush hour to select and record the victims. Compared to the morning when most people report to their work by nine o’clock, the after-work commute times tend to vary, ranging from five to eight in the evening. The less-crowded train cars and stations facilitated surreptitious recording offenses by creating a suitable distance between the offenders and the victims. Additionally, the offenders preferred the stairs and the escalators located in high-traffic areas. Where large crowds of passengers walked through to transfer lines were usually favored by the offenders due to abundant naturally-created blind spots that allowed them to act stealthily.

Prior to recording, the offenders familiarized themselves with the surrounding areas. They were also shown to look for the escalators and the stairways made of opaque material that were built on a steep slope, as well as the areas not monitored well by CCTV in an effort to minimize the risk of failure and apprehension.

As for the tools used, older smartphones whose data were known to be difficult to recover by forensic technologies were preferred. Applications that disabled the shutter sounds were often used and in some cases, an endoscope was connected to the phone to photograph or film in a further cunning manner. Additionally, various forms of hidden camera such as the button camaras under shoelaces and shirts as well as pen and watch cameras were widely used. Furthermore, as in some cases of sexual harassment, the offenders waited and searched for a person to whom they were attracted or an intoxicated pedestrian around the crime scene (i.e., stairways or escalators).

#### 4.2.2. Criminal Event Stage

The offenders exhibited two main behavioral patterns. First, they walked up or stayed two to three steps behind the victims to create a proper distance for recording. Before proceeding to take photos or film the victims, the offenders scanned the surrounding areas for CCTV and police patrol stakeouts. The recording usually began around the halfway up the stairs or escalators. In the cases in which the cameras were hidden underneath shoe laces and the offense was committed on the escalators, the offenders placed the foot with the camera one step higher than the other foot so the camera was close and directed toward inside the victims’ skirts. Hiding the cameras in a hand-held or a shopping bag was another popular method. If the victims did not notice being recorded, the offending continued until the they descended the escalators or climbed to the top of the stairs.

#### 4.2.3. Post-Offense Stage

**Figure 3:**
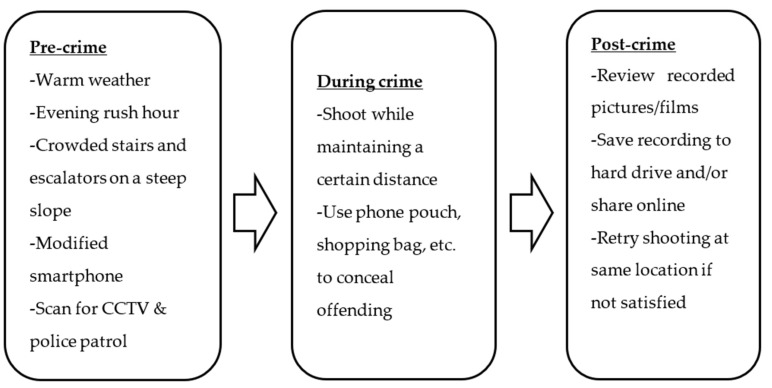
Surreptitious Shooting Crime Script. Remove paragraph symbols.

In the post-offense phase, the offenders reviewed the files immediately after moving to a quiet area. They then saved what had been shot to the hard drive and/or shared it with their accomplices and others to upload to the Internet or distribute among the private message group members. If not satisfied with the end results, the offenders returned to the same escalator or stairways to repeat what they had done. Because the offenders were known to return to the same location, the officers reported that the police closely monitored suspicious individuals that appeared at a specific escalator or stairs continuously (see Figure 3 for a summary of the entire crime-commission process).

## 5. Discussion

The current research examined the behavioral patterns of the subway sex offenders who had committed harassment and surreptitious recording in the city of Seoul, South Korea. The CSA results identified notable routines involved in the crime-commission process for each offense. Of different forms of sexual harassment [21], a focus was non-consensual physical contact which was shown to be committed mostly in crowded cars during morning and evening rush hours. The offenders selected the targets and scanned for CCTV and law enforcement presence before boarding the train. Once inside, the harassers stood behind or face-to-face with the targets in close proximity.

The most common ways of making physical contact involved rubbing hands and genitals against the buttocks and thighs as well as blowing into the ears of the victims. If there were no responses from the harassed, the offenders continued and became more aggressive. In the last phase, the offenders disembarked the train through a planned exit or moved to another car to find the next victims. If confronted by the victims, the harassers either fled or denied offending.

The finding that the offenders preferred the morning and evening rush hour was in accord with the results of existing research. Since overcrowding facilitates sex crimes [64,65], morning and evening times when people are going to or returning from work are identified as the most-risky periods [23,25].

Density was also an important factor for surreptitious recording. However, the incidents were more prevalent in the evening hours during which the stations were not as crowded as in the morning, which created a suitable distance between the offenders and the targets for recording. Surreptitious recording did not occur as frequently as harassment, which could be explained by the impact of the weather. Particularly, the recording offenses increased in warmer months when female passengers were in lighter clothing such as short skirts. Additionally, the need for tools including modified smartphone and/or hidden camera makes surreptitious recording a skilled crime that could require more learning and practice than harassment.

The voyeurs scanned the surrounding areas for CCTV and police patrol before started recording and often used an item such as a pouch or a bag to conceal their actions. One noteworthy pattern in the post-crime stage was reviewing of recorded files and re-attempting at shooting if not satisfied. The offenders were found to save the files to an external drive and shared them with the members of online communities. As in other subcultural communities (e.g., [66,67]), they may find a sense of membership and satisfaction by exchanging the recorded files. The online communities could also offer information regarding avoiding detection by law enforcement and decreasing the risk of apprehension [66]. However, discussion of the sex offender subculture in cyberspace is beyond the scope of the current research so future studies should explore this line of inquiry. The following limitations should be considered before discussing the theoretical and practical implications based on the findings. First, the results drawn from the small sample comprised of thirty-five case files and twelve law enforcement interviewees cannot be generalized to a larger population of the subway sex offenders in the country. Moreover, despite the authors’ effort to consult different types of data (i.e. crime files, official statistics, and officer interviews), the interview participants’ subjective perspectives and potential distortions due to memory errors could have biased the results [68]. Therefore, offender interview (both those who have been and have not been prosecuted) could provide further insights into the modus operandi as well as the effective crime prevention measures. Furthermore, the passenger perceptions regarding security could contribute to creating a safer travel environment. Lastly, conducting experiments to evaluate the effects of the crime prevention programs could help implement policies founded on empirical evidence.

Despite the limitations, the current research contributes to the existing literature as the first systematic analysis of the patterns and the modus operandi of the two most prevalent subway sex offenses in Seoul, South Korea. Specifically, the authors were able to gain detailed understanding of sexual harassment and surreptitious recording by employing CSA. Approaching the offenses as a process, each stage of the offenses was analyzed and the intervention points could be identified from prior to committing the crimes to the aftermath [47,69].

The findings suggest that RAT offers a robust theoretical framework for explaining subway sex offense victimization. The number of incidents for both types of crime increased when the offenders and the suitable targets converged spatio-temporally in the absence of capable guardians. The opportunities for harassment and voyeurism were created by passengers’ daily routines, especially the morning and evening commute which provided numerous potential targets for the offenders.

It was also shown that that offender decision making was crime-specific and each phase of the crime-commission process involved cost-benefit analysis [43,70]. Based on Clarke and Eck’s [71] problem analysis triangle, the authors suggest the following prevention measures (Table 2) utilizing the Situational Crime Prevention (SCP) techniques [72]. Because SCP aims to reduce criminal opportunities within the framework of RAT [25], the strategies emphasize reducing the spatial-temporal convergence of a suitable target and a motivated offender in the absence of a capable guardian.

For both sexual harassment and surreptitious recording, utilizing place managers to a greater extent and strengthening formal surveillance could increase the risks for the offenders. To illustrate, charging additional subway staff with patrolling the stations and the passenger cars to detect and communicate suspicious activities to the authorities will increase guardianship. Moreover, additional CCTV surveillance inside the cars and around the station could deter the motivated offenders. Increasing the amount of police patrol (both uniform and plain clothes) when and where the offenses are concentrated would be an effective strategy as well. In a study that has examined sexual victimization of college students in public transportation, most participants have identified similar SCP techniques as recommended measures, namely plain-clothes patrolling, more staff presence, and additional CCTV [32].

Furthermore, posting signs in the stations and the passenger cars to alert conscience (i.e. remove excuses) could deter motivated offenders. For instance, boards displaying slogans such as “unwanted touching is a sexual assault” and “you will be prosecuted” could not only increase the likely offenders’ perceived risk but also the passenger awareness about the issue of sexual harassment in the subway.

Research shows that most cases of sexual victimization are not reported to law enforcement [32]. It is possible that the victims’ reluctance to notify the authorities is a result of a lack of formal sources or support and possible victim blaming [73,74]. To promote public awareness and victimization reporting, efforts must be directed toward informing the public that subway sex offenses are serious and prevalent issues. Posters and boards around subway stations and inside the cars containing information about the offenses, ways to reduce victimization, and the resources available to the victims such as the numbers to call could increase reporting rates [23,65]. A broader scale of social campaigns as well as legal reforms spearheaded by the central government could create additional sources and programs to support the victims and allow law enforcement to address the crimes effectively [75].

Improving lighting in and around the stations could prevent victimization by assisting natural surveillance [32,34]. The offenders were reported to search for the stairways and the escalators whose sides were built with non-transparent material. Therefore, replacing the opaque material with glass can improve visibility and increase the risks for the voyeurs. It may also be a viable plan to close the escalators at the hot spots during the hot times to reduce the opportunities for recording [76].

In order for the crime prevention measures for the sex offenses committed in public transportation to have a long-term impact, a multifaceted approach with a wide range of intervention points should be considered [19,77]. Therefore, aside from educating the passengers who are potential victims of the subway offenses, police officers must receive proper training so they are able to handle the cases appropriately. Particularly, the officers need to be informed about the seriousness of the offenses as well as their impact on the victims. It has been noted that the prevention measure supported the most by the passengers is the authorities taking sex offense-related complaints seriously [32]. Continuous effort must be made to inform the law authorities about the nature and the state of the subway sex offenses.

Lastly, cooperation between the Seoul Metro and the police could be enhanced by establishing a guideline for preventing and responding to the sex offenses. If implemented successfully, such partnership could benefit from synergy effects [78]. The two organizations could participate in joint training and education programs to discuss the missions and the values that they share and the ways to take a collaborative crime control approach to the subway sex offenses.

## 6. Conclusions

Subway sex offenses are a prevalent and serious issue in South Korea. Unfortunately, no systematic research has been conducted on the subject. In an attempt to fill this gap, the current research employed CSA and examined sexual harassment and surreptitious recording, the two most common types of subway sex crime in Seoul, South Korea.

The findings showed that there were a few notable differences in the situational contexts in which the two offenses were committed, demonstrating that classifying the subway crimes into one category [4,9,24,31,37,38] is not a reasonable approach. The authors also confirmed that both the harassers and the voyeurs made their decisions rationally at each phase of the crime-commission process, exhibited by the efforts invested to maximize success and minimize failure. It was also found that situational factors such as passenger density and traffic volume created opportunities for the offenders who employed strategies such as scanning for cameras and law enforcement presence and escaping through planned routes.

Based on the results, the authors suggest SCP be at the foundation of the prevention response measures for the offenses. The SCP techniques including utilizing place managers, strengthening formal surveillance, assisting natural surveillance, and alerting conscience can help to reduce criminal opportunities while increasing the offenders’ perceived risks [79] and raise public awareness. Aside from the SCP techniques, systematic cooperation between the Seoul Metro and the Seoul Metropolitan Police Agency could realize effective crime prevention and response programs.

Furthermore, policymakers could attempt to revive discontinued programs such as women-only subway cars after a thorough examination of the demand for it as well as its impact on crime reduction. Based on extensive evidence of higher victimization risks and fear of crime among female passengers as well as their distinct travel patterns, women-only passenger cars could be a supplement to the aforementioned crime prevention strategies if implemented after a careful investigation of the passengers’ attitudes and its effectiveness.

## Figures and Tables

**Table 1:** Seoul Metro Crime Incidents (2015-2019) [6].

Year	Total	Surreptitious Recording	Sexual Harassment	Theft	Other
2015	3040	1040	779	825	396
2016	2752	689	799	727	537
2017	3082	717	1094	678	593
2018	2599	474	754	635	736
2019	2755	378	828	694	855

**Table 2:** Situational Crime Prevention Techniques for Preventing Subway Sex Offenses.

Increase the Risks	Remove Excuses
Assist natural surveillance Improved lighting Transparent stairways and escalators	Alert conscience “Unwanted touching is a sexual assault” “You will be prosecuted” Subway sex crime information boards and posters
Utilize place managers Subway staff patrol Additional CCTV	
Strengthen formal surveillance Hot spots and hot times police patrol	

## References

[1] Ryan S. (1999). Property values and transportation facilities: Finding the transportation—Land use connection. J. Plan. Lit..

[2] Mahler J. (2018). The Case for the Subway: It Built the City. Now, No Matter the Cost—At Least $100 Billion—The City Must Rebuild It to Survive. https://www.nytimes.com/2018/01/03/magazine/subway-new-york-city-public-transportation-wealth-inequality.html.

[3] Wachter S.M., Gillen K.C. Public Investment Strategies: How They Matter for Neighborhoods in Philadelphia.

[4] Shim H.J., Shin S.R., Cho Y.O. (2017). Do routine activities among college students affect their sex crime victimization in subway. J. Korean Police Assoc..

[5] Seoul Metro: Message from the CEO. http://www.seoulmetro.co.kr/en/page.do?menuIdx=649.

[6] Korean National Police Agency (2020). Prevention of Sex Crimes on the Subway in Seoul.

[7] Fairchild K., Rudman L.A. (2008). Everyday stranger harassment and women’s objectification. Soc. Justice Res..

[8] Horii M., Burgess A. (2012). Constructing sexual risk: ‘Chikan’, collapsing male authority and the emergence of women-only train carriages in Japan. Healthrisk Soc..

[9] Loukaitou-Sideris A., Fink C. (2008). Addressing women’s fear of victimization in transportation settings: A survey of U.S. transit agencies. Urban Aff. Rev..

[10] Krahe B. (2005). Cognitive coping with the threat of rape: Vigilance and cognitive avoidance. J. Pers..

[11] Warr M. (1985). Fear of rape among urban women. Soc. Probl..

[12] Maslow A.H. (1943). A theory of human motivation. Psychol. Rev..

[13] Loukaitou-Sideris A. (2014). Fear and safety in transit environments from the women’s perspective. Secur. J..

[14] Gillespie A. (2008). “Up-skirts” and “down-blouses”: Voyeurism and the law. Crim. Law Rev..

[15] Madan M., Nalla M.K. (2016). Sexual harassment in public spaces: Examining gender differences in perceived seriousness and victimization. Int. Crim. Justice Rev..

[16] Seoul Foundation of Women and Family (2019). 2018 Seoul Gender Sensitivity Statistics.

[17] Lee J.L. (2013). An analysis of the current state of subway sex offenses by type and effective countermeasures. Korean Crime Psychol. Study.

[18] Cornish D.B. (1994). Crimes as scripts. Proceedings of the International Seminar on Environmental Criminology and Crime Analysis (University of Miami, Coral Gables, Florida, May 1993).

[19] Gekoski A., Gray J.M., Horvath M.A., Edwards S., Emirali A., Adler J.R. (2015). ’What Works’ in Reducing Sexual Harassment and Sexual Offences on Public Transport Nationally and Internationally: A Rapid Evidence Assessment.

[20] Lahsaeizadeh A., Yousefinejad E. (2012). Social aspects of women’s experiences of sexual harassment in public places in Iran. Sex. Cult..

[21] Tripathi K., Borrion H., Belur J. (2017). Sexual harassment of students on public transport: An exploratory study in Lucknow, India. Crime Prev. Community Saf..

[22] Dunckel-Graglia A. (2013). Women-only transportation: How “pink” public transportation changes public perception of women’s mobility. J. Public Transp..

[23] Gautam N., Sapakota N., Shrestha S., Regmi D. (2019). Sexual harassment in public transportation among female student in Kathmandu valley. Risk Manag. Healthc. Policy.

[24] Ceccato V., Nalla M.K. (2020). Crime and Fear in Public Places: Towards Safe, Inclusive and Sustainable Cities.

[25] Cohen L.E., Felson M. (1979). Social change and crime rate trends: A routine activity approach. Am. Sociol. Rev..

[26] Newton A.D. (2014). Crime on Public Transport.

[27] American Psychological Association Frotteurism. APA Dictionary of Psychology.

[28] American Psychological Association Voyeurism. APA Dictionary of Psychology.

[29] Firestone P., Kingston D.A., Wexler A., Bradford J.M. (2006). Long-term follow up of exhibitionists: Psychological, phallometric, and offense characteristics. J. Am. Acad. Psychiatry Law.

[30] Krueger R.B., Kaplan M.S., Laws D.R., O’Donohue W.T. (2008). Frotteurism: Assessment and Treatment. Sexual Deviance: Theory, Assessment, and Treatment.

[31] Lee H.S. (2014). A study on subway crime prevention utilizing CPTED. Police Acad. Conf..

[32] Natarajan M., Schmuhl M., Sudula S., Mandala M. (2017). Sexual victimization of college students in public transport environments: A whole journey approach. Crime Prev. Community Saf..

[33] Neupane G., Chesney-Lind M. (2014). Violence against women on public transport in Nepal: Sexual harassment and the spatial expression of male privilege. Int. J. Comp. Appl. Crim. Justice.

[34] Smith M.J. (2008). Addressing the security needs of women passengers on public transport. Secur. J..

[35] Pedersen L. (2020). Moving bodies as moving targets: A feminist perspective on sexual violence in transit. Open Philos..

[36] Valentine G. (1989). The geography of women’s fear. Area.

[37] Kalms N., Korsmeyer H. (2017). Gender Makes a World of Difference for Safety on Public Transport. https://www.smh.com.au/lifestyle/gender-makes-a-world-of-difference-for-safety-on-public-transport-20170718-gxd8ee.html.

[38] Ceccato V., Paz Y. (2017). Crime in São Paulo’s metro system: Sexual crimes against women. Crime Prev. Community Saf..

[39] Hwang J.T. (2003). A Study on the Crime in Subway.

[40] Jeffery C.R. (1997). Crime Prevention through Environmental Design.

[41] Lee K.H., Shin Y.J., Um A.Y., Par J.E., Lee J.M., Choi S.H. (2014). A study of crime-causing factors in subway stations through environmental design for crime prevention: Focused on cases of Seoul station and Sinderim station. J. Korean Soc. Des. Cult..

[42] Roh S.H., Kim H.K. (2012). Preventing subway crimes: Focusing on sexual offenses in the Seoul metropolitan subway. J. Korea Creat. Content Assoc..

[43] Cornish D.B., Clarke R.V. (1986). Introduction. The Reasoning Criminal: Rational Choice Perspectives on Offending.

[44] Cornish D.B., Clarke R.V., Piquero A.R., Tibbetts S.G. (2002). Analyzing organized crimes. Rational Choice and Criminal Behaviour: Recent Research and Future Challenges.

[45] Cornish D.B., Clarke R.V. (1994). The procedural analysis of offending and its relevance for situational prevention. Crime Prevention Studies.

[46] American Psychological Association Schema. APA Dictionary of Psychology.

[47] Chiu Y.N., Leclerc B., Townsley M. (2011). Crime script analysis of drug manufacturing in clandestine laboratories: Implications for prevention. Br. J. Criminol..

[48] Tedeschi J.T., Felson R.B. (1994). Violence, Aggression, and Coercive Actions.

[49] Levi M., Soudijn M. (2020). Understanding the laundering of organized crime money. Crime Justice.

[50] Bullock K., Clarke R.V., Tilley N. (2010). Situational Prevention of Organised Crime.

[51] Masys A.J., Masys A.J. (2016). Disrupting terrorist and criminal networks: Crime script analysis through DODAF applications. Exploring the Security Landscape.

[52] Morselli C., Roy J. (2008). Brokerage qualifications in ringing operations. Criminology.

[53] Lacoste J., Tremblay P., Smith M.J., Cornish D.B. (2003). Crime innovation: A script analysis of patterns in check forgery. Crime Prevention Studies.

[54] Beauregard E., Leclerc B. (2007). An application of the rational choice approach to the offending process of sex offenders: A closer look at the decision-making. Sex. Abus. J. Res. Treat..

[55] Beauregard E., Rebocho M.F., Rossmo D.K. (2010). Target selection patterns in rape. J. Investig. Psychol. Offender Profiling.

[56] Tompson L., Chainey S. (2011). Profiling illegal waste activity: Using crime scripts as a data collection and analytical strategy. Eur. J. Crim. Policy Res..

[57] Hutchings A., Holt T.J. (2015). A crime script analysis of the online stolen data market. Br. J. Criminol..

[58] Choi K., Lee J.L., Chun Y.T. (2017). Voice phishing fraud and its modus operandi. Secur. J..

[59] Leclerc B., Wortley R., Smallbone S. (2011). Getting into the script of adult child sex offenders and mapping out situational prevention measures. J. Res. Crime Delinq..

[60] Beauregard E., Proulx J., Rossmo K., Leclerc B., Allaire J.F. (2007). Script analysis of the hunting process of serial sex offenders. Crim. Justice Behav..

[61] Korean Statistical Information Service Population. https://kosis.kr/statisticsList/statisticsListIndex.do?menuId=M_01_01&vwcd=MT_ZTITLE&parmTabId=M_01_01#SelectStatsBoxDiv.

[62] Yin R.K. (2003). Applications of Case Study Research.

[63] Gibbs G. (2007). Analyzing Qualitative Data.

[64] Brantingham P. (1995). Criminality of place: Crime generators and crime attractors. Eur. J. Crim. Policy Res..

[65] Clark S.K., Jeglic E.L., Calkins C., Tatar J.R. (2016). More than a nuisance: The prevalence and consequences of frotteurism and exhibitionism. Sex. Abus..

[66] Blevins K.R., Holt T.J. (2009). Examining the virtual subculture of johns. J. Contemp. Ethnogr..

[67] Holt T.J., Blevins K.R., Burkert N. (2010). Considering the pedophile subculture online. Sex. Abus..

[68] Porter L.E. (2008). Using archival data and multidimensional scaling to explore leadership: Examples from group crime. Issues Forensic Psychol..

[69] Levi M., Maguire M. (2004). Reducing and preventing organised crime: An evidence-based critique. Crime Law Soc. Chang..

[70] Cornish D.B., Clarke R.V. (2008). The rational choice perspective. Environ. Criminol. Crime Anal..

[71] Clarke R.V., Eck J.E. (2005). Crime Analysis for Problem Solvers in 60 Small Steps.

[72] Clarke R.V. (1997). Situational Crime Prevention: Successful Case Studies.

[73] Ullman S.E. (1999). Social support and recovery from sexual assault: A review. Aggress. Violent Behav..

[74] Ullman S.E., Filipas H.H. (2001). Correlates of formal and informal support seeking in sexual assault victims. J. Interpers. Violence.

[75] Baumer E.P. (2004). Temporal Variation in The Likelihood of Police Notification by Victims of Rapes 1973–2000 (NCJ 207497).

[76] La Vigne N.G. (1997). Visibility and Vigilance: Metro’s Situational Approach to Preventing Subway Crime.

[77] Clarke R.V., Newman G.R. (2006). Outsmarting the Terrorists.

[78] Johnston L., Shearing C. (2003). Governing Security: Explorations in Policing and Justice.

[79] Lab S.P. (2007). Crime Prevention: Approaches, Practices and Evaluation.

